# Detection of *Coxiella burnetii* and characterisation by multiple-locus variable-number tandem repeat analysis in bovine bulk tank milk samples

**DOI:** 10.17221/87/2022-VETMED

**Published:** 2023-05-25

**Authors:** Berna Yanmaz, Ediz Kagan Ozgen

**Affiliations:** ^1^Department of Public Health, Faculty of Veterinary Medicine, Burdur Mehmet Akif Ersoy University, Burdur, Turkiye; ^2^Erzurum Veterinary Control Institute, Republic of Turkiye Ministry of Agriculture and Forestry, Erzurum, Turkiye

**Keywords:** genotyping, Q fever, raw milk, Turkiye

## Abstract

*Coxiella burnetii* is the aetiological agent of Q fever, which is highly prevalent in Turkiye, but information on the genetic profiles of the bacterium is limited. This study aimed to investigate the presence of *C. burnetii* in bovine bulk tank milk (BTM) samples by polymerase chain reaction (PCR) and to investigate the genotypes by means of multiple-locus variable-number tandem repeat analysis (MLVA). A total of 25 markets that sold raw cow’s milk were analysed by conventional PCR analysis. An MLVA analysis was performed at six loci, namely MS23, MS24, MS27, MS28, MS33, and MS34, to determine the genotypic variations of *C. burnetii* found in the positive DNA samples. The DNA of *C. burnetii* was detected in 16% of the BTM samples. The *C. burnetii* strains identified in the bovine milk samples collected in this study were found to belong to the same genotypic group as those detected in the bovine milk samples gathered in Greece. As a result, both the presence and genotyping studies of *C. burnetii* on the BTM samples in Turkiye will contribute to the determination of the geographical distribution of the agent.

Q fever is a global zoonotic disease caused by *Coxiella burnetii*, an obligate Gram-negative bacterium, which can infect various animal species and humans (Eldin et al. 2017). Ruminants are usually asymptomatic carriers of *C. burnetii* and are considered a source of infection for humans (Ceglie et al. 2015). *C. burnetii* is transmitted to humans mainly by inhalation of bacteria-contaminated aerosols and following direct contact with infected animals or their products (Pexara et al. 2018a).

The most common method to detect *C. burnetii* in milk is a real time polymerase chain reaction (PCR) analysis (Jodelko et al. 2021). The method can be utilised for either an animal’s individual milk samples or bulk tank milk (BTM) (Rabaza et al. 2021). Analysis of BTM samples is practical and relatively inexpensive to investigate for *C. burnetii* at the farm level and estimate their regional prevalence (Pexara et al. 2018b). Moreover, due to the discontinuous shedding pattern of *C. burnetii* (Guatteo et al. 2007), BTM is more preferred than an individual milk analysis.

The incidence of *C. burnetii* has increased rapidly in many regions in Turkiye (Bagatir et al. 2021). Erzurum is an eastern city of Turkiye where livestock exchange occurs frequently and animal husbandry is intensive. Although molecular prevalence rates of *C. burnetii* in sheep and cattle have been reported from many regions in Turkiye (Can et al. 2015; Gulmez Saglam and Sahin 2016), to the best of our knowledge, there is limited data on the molecular prevalence of *C. burnetii* in the BTM in Turkiye (Can et al. 2015).

Genotyping is useful for analysing the available circulation of *C. burnetii* strains and finding the potential relationship between the genotypes and the virulence of the strains (Arricau-Bouvery et al. 2006). Multiple-locus variable-number tandem repeat analysis (MLVA) is useful for identifying and genotyping *C. burnetii* isolates (Pinero et al. 2015). By using the MLVA method, the genotypic relatedness can be revealed from the same DNA sample of molecularly identified strains. Thus, the necessity of isolating the bacteria in a culture is eliminated (Cumbassa et al. 2015). Various studies from different countries have identified the *C. burnetii* MLVA genotypes that infect dairy cattle herds (Mioni et al. 2019; Bauer et al. 2021; Hemsley et al. 2023). A recent study in the Northeast Anatolia Region of Turkiye showed that cattle genotypes play an active role in the transmission of *C. burnetii* to humans (Ozgen et al. 2022). The objectives of this study were: 1) to demonstrate the presence of *C. burnetii* in the BTM in Erzurum province, where cattle breeding is high, 2) to investigate the *C. burnetii* MLVA genotypes found in the DNA-positive PCR samples. We hypothesise that the MLVA profiles of the *C. burnetii* isolates from different geographic regions will exhibit genetic diversity, indicating the presence of multiple strains of the bacterium. This information could provide valuable insights into the epidemiology and transmission dynamics of *C. burnetii* in dairy cattle populations, and potentially contribute to the development of control measures to reduce the risk of human infection.

## MATERIAL AND METHODS

### Sample collection

BTM samples were randomly collected from 25 markets located in the central region of Erzurum, in the Northeast of Turkiye, which sell local food products, and the convenience sampling method was used for this study. Samples were collected in 50 ml sterile plastic tubes and then immediately transferred to the laboratory and stored at +4 °C until required for further analysis. The BTM samples were collected between March and April 2022. The Erzurum Veterinary Control Institute Animal Experiments Local Ethics Committee approved the study with a protocol No. 2022/89.

### Processing of the milk and DNA extraction

The cream was separated from the milk samples prior to the DNA extraction. In order to separate the cream, the milk was transferred into 1 ml sterile microcentrifuge tubes and then centrifuged at 13 600 × *g* for 60 minutes. After the cream layer had been separated, the remaining pellet was washed twice with phosphate-buffered saline (PBS). After washing, the pellet was diluted with 200 μl of PBS (Berri et al. 2000). To extract the DNA, we used a QIAamp cador Pathogen Nucleic Acid Extraction Kit (Qiagen, Courtaboeuf, France). After extraction, 50 μl of eluate was collected for each sample. The quality of the DNA was evaluated by measuring the optical density (OD) 260/280 ratio (≅ 1.8) and the OD 260/230 ratio (≅ 2.02) using a NanoDrop 2000 spectrophotometer (Thermo Fisher Scientific, Waltham, USA). Samples with acceptable ratios were deemed suitable for further analysis. The DNA samples were stored at –20 °C until the PCR and MLVA analyses. The *C. burnetii* Nine Mile phase strain was used as the positive control in the DNA extraction, PCR amplification, and MLVA analysis.

For the PCR amplification, a Labcycler Gradient (SensoQuest, Göttingen, Germany) thermal cycler was used. A total volume of 25 μl was prepared, containing 12.5 μl of the Hotstart Master Mix (Qiagen, Hilden, Germany), 1 μl of each primer, 3 μl of the extracted DNA samples, and 7.5 μl of deionised distilled water. A touchdown PCR program was employed, consisting of an initial denaturation step at 95 °C for 15 min, followed by 6 cycles of denaturation at 94 °C for 30 s, annealing at 66 °C with a decrease of 1 °C per cycle for 1 min, and extension at 72 °C for 1 minute. Subsequently, 40 cycles of denaturation at 94 °C for 30 s, annealing at 61 °C for 30 s, and extension at 72 °C for 1 min were performed. A final extension was performed at 72 °C for 5 minutes.

### Identification of *Coxiella burnetii*

**Figure 1:**
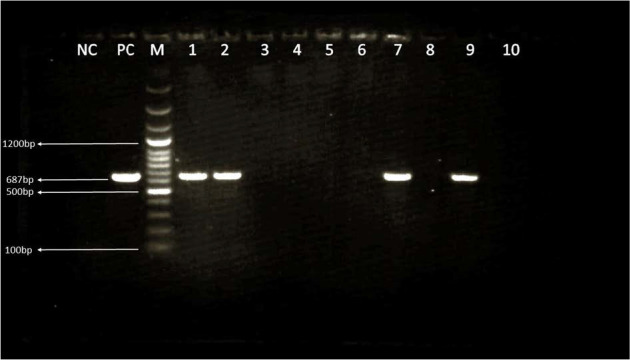
The agarose gel image of the *C. burnetii* positive bulk tank milk samples The reference indicator (M = ladder, 100–3 000 bp), NC (negative control), PC (positive control), 1^st^, 2^nd^, 7^th^, and 9^th^ places are the 687 bp bands belonging to the positive samples

In terms of the identification of *C. burnetii*, a conventional PCR analysis was performed using the *IS1111* gene region-specific Trans1 and Trans2 primers (Berri et al. 2000). The obtained amplicons were identified in 1.5% agarose gel by electrophoresis. The gel was prepared by mixing 1.5% agarose with 6x loading dye, and 10 μl of the PCR product was loaded into each well. One well was loaded with a 100–3 000 bp DNA ladder, one well with a positive control PCR amplicon, and one well with a negative control PCR amplicon. The electrophoresis was performed at 60 volts and 400 milliamperes for 90 minutes. The PCR products were then visualised using an electrophoresis power station. Agarose gel was placed in the transilluminator and imaging was performed under UV light through the GeneLine ImageSource Software program (Spectronics Corporation, USA). *C. burnetii* was considered positive if amplicons were detected in the agarose gel with a 687 bp DNA fragment (Figure 1).

### MLVA analysis

An MLVA analysis was performed at six loci, namely MS23, MS24, MS27, MS28, MS33, and MS34, to determine the genotypic variations of the *C. burnetii* found in the positive DNA samples (Klaassen et al. 2009; Tilburg et al. 2012). After the reactions were prepared in 20 μl volumes, 10 μl of the HotStarTaq Master Mix (Qiagen, Courtaboeuf, France), 6 μl of deionised distilled water, 0.5 μM of primer, and 2 μl of the sample DNA were added to 0.2 ml tubes and then heated in a thermal cycler at 95 °C. After 15 min of initial denaturation, 40 cycles were performed at 95 °C for 30 s, at 60 °C for 45 s, and at 72 °C for 90 seconds. With regard to the final elongation phase, the denaturation was applied at 72 °C for 7 minutes. The optimisation of the test was performed using a *C. burnetii* Nine Mile Phase I DNA sample. A QIAxcel Advanced System (Qiagen, Courtaboeuf, France) was used to perform the capillary electrophoresis in order to determine the locus sizes. The number of repeats was then calculated for each locus (Racic et al. 2014).

The MLVA profiles of the *C. burnetii* identified in the positive samples were compared with the MLVA bank database from France, Germany, Slovakia, Japan, Hungary, the Netherlands, Spain, the United Kingdom, Switzerland, Russia, Saudi Arabia, Qatar, Portugal, Italy, Poland and Greece (MLVA Bank 2022). A nexus file concerning the closeness between the strains was prepared using the unweighted pair group and mathematical averaging method based on the Hamming/P Distance Similarity Index using the Past v4.09 (Palaeontological Association, Norway) software program for the MLVA profiles obtained from the database.

## RESULTS

The DNA of* C. burnetii* was detected in 16% of the BTM samples obtained from the sellers of the local raw cow’s milk in Erzurum. During the optimisation of the MLVA, we found that the *C. burnetii* Nine Mile Phase I positive control DNA sample contained 9, 27, 4, 6, 9, and 5 repeats, respectively, at the MS23, MS24, MS27, MS28, MS33, and MS34 loci. The numbers of repeats detected in the MS23, MS24, MS27, MS28, MS33, and MS34 loci as a result of the MLVA analysis of the DNA samples determined to be positive *C. burnetii* are shown in Table 1.

**Table 1:** MLVA results from the *C. burnetii* positive bovine bulk tank milk samples

Code of positive samples	MS23	MS24	MS27	MS28	MS33	MS34
CbCM01	NA	NA	2	3	NA	NA
CbCM02	NA	NA	3	3	NA	NA
CbCM03	NA	NA	3	3	NA	NA
CbCM04	NA	NA	2	3	NA	NA

CbCM = *Coxiella burnetii* cattle milk; NA = not amplified

**Figure 2:**
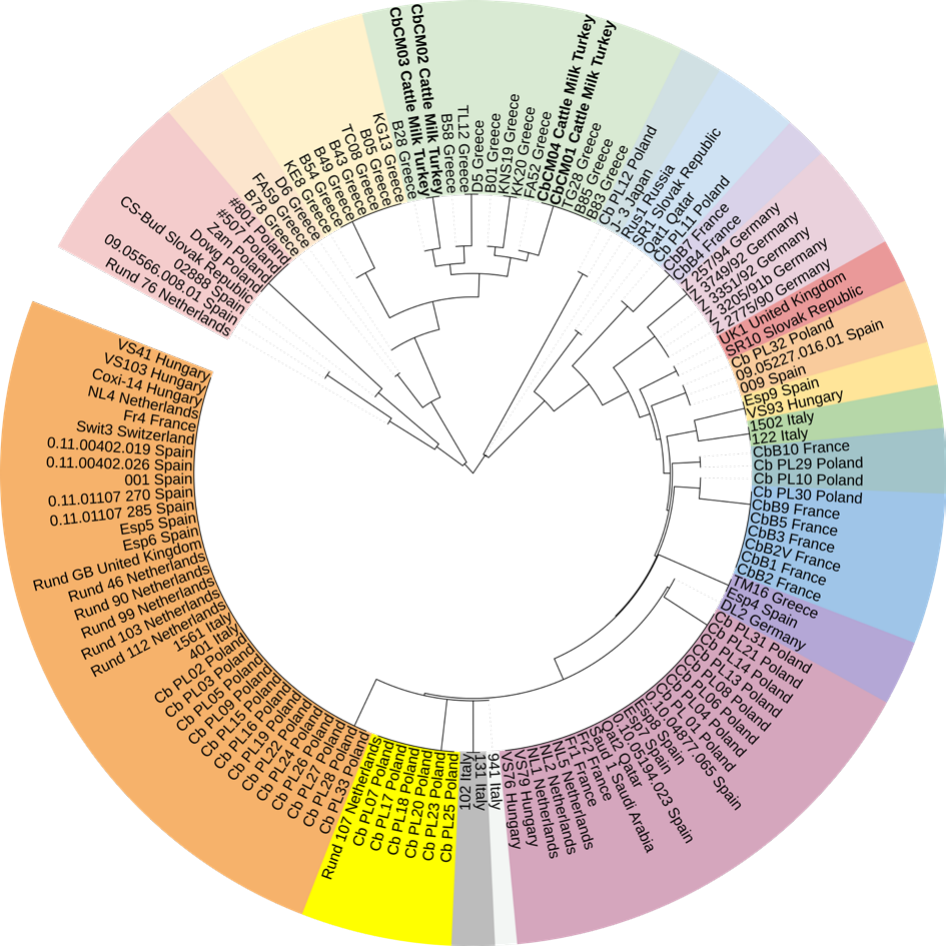
A circular dendrogram was created to represent the genotypes of *C. burnetii* found in the bulk tank milk samples, and to compare them with the genotypes present in the MLVA Bank for the Microbes Genotyping database A total of 131 genotypes from 16 different countries, primarily European countries, were compared, resulting in the formation of 20 genotypic groups. The genotypes identified in this study are highlighted in bold characters. Notably, our study revealed that the genotypes detected in our samples were clustered together with those reported from Greece

A circular dendrogram (Figure 2) was generated by comparing the MLVA genotype structures of the *C. burnetii* DNA detected in the milk samples with those from previous studies (MLVA Bank 2022). We compared a total of 131 bovine *C. burnetii* MLVA profiles from 16 different countries and identified 20 genotypic groups. The *C. burnetii* detected in the bovine milk samples collected in this study were found to be localised within the same genotypic group as those detected in the bovine milk samples from Greece. This particular genotypic group was also observed to have the highest number of *C. burnetii* among all the identified genotypic groups.

## DISCUSSION

In this study, the DNA of* C. burnetii* was detected in 16% of the BTM samples collected from the sellers of the local raw cow’s milk in Erzurum. This study is one of the first to analyse the detection of *C. burnetii* DNA by PCR in BTM samples obtained from cattle in Turkiye. Previous studies conducted in Turkiye have reported PCR positivity levels for *C. burnetii* in bovine milk samples ranging from 1.42% to 10% (Can et al. 2015; Gulmez Saglam and Sahin 2016). Furthermore, it has been determined that the global molecular prevalence of *C. burnetii* in BTM samples is 37% (Rabaza et al. 2021), which is considerably higher than the positivity rate identified in this study. The observed disparity in the results might be associated with the climatic conditions of the sampling location, as Erzurum is a city located in the Northeast Anatolia region of Turkiye, which is known for its cold weather and has an altitude of 1 850 meters above sea level. The lower temperatures in the city may have caused a reduction in the shedding of *C. burnetii*. A previous study reported that the shedding of *C. burnetii* tends to increase during hot and dry weather conditions, likely due to the airborne transmission of contaminated dust particles (Nusinovici et al. 2015). Examinations of BTM samples have shown a global prevalence of 37% (Rabaza et al. 2021), but the studies also indicated that the prevalence can vary widely, ranging from 10.7% to 76.9% (USDA APHIS 2011; Boroduske et al. 2017). The mechanism of transmission for this agent is associated with contact between animals and the agent load within the herd (Rabaza et al. 2021). The observed variations may be due to differences in the sampling periods and with the used analytical methods (Gulmez Saglam and Sahin 2016).

In the MLVA analysis of *C. burnetii* detected in this study, a partial genotype was determined despite high DNA loading. Studies conducted on milk tank samples have reported that mixing milk from positive and negative individuals can lead to reduced DNA concentrations, resulting in partial genotyping in the MLVA analysis (Tilburg et al. 2012). Another reason for the lower DNA concentration could be related to the insertions or deletions in the genome, which may prevent the detection of repeats in a locus (Sidi-Boumedine et al. 2015). In addition, it has been reported that there could be amplification failures or unexpected amplifications at the MS23 and MS33 loci (Sidi-Boumedine et al. 2015).

The current research identified partial genotypes with 2-3 and 3-3 repeat numbers at the MS27 and MS28 loci, respectively. By comparing 131 *C. burnetii* MLVA samples obtained from 16 different countries, which are available in the MLVA Bank 2022, the partial MLVA genotype profiles identified in this study were found to belong to the same group as those reported by Kalaitzakis et al. (2021). However, a complete comparison could not be made due to the partial nature of the genotypes. When examining the genotypes MS27 and MS28 2-3 from the MLVA Bank for the Microbes Genotyping database, they were found to be present in cattle, sheep, goats, and humans from Spain, France, Portugal, Iran, and Morocco. MS27 consists of a total of 9 genotypes between 43 and 51 of the MS28 2-3 genotype profile to the MLVA6Nijmegen genotype number, as reported by Arricau-Bouvery et al. (2006), Astobiza et al. (2012), and Santos et al. (2012). In the MLVA Bank for the Microbes Genotyping database, there are 15 different genotypes with 3-3 repeats at the MS27 and MS28 loci, identified from cattle, sheep, goats, and humans from various countries such as the Netherlands, France, Austria, Spain, Germany, Italy, Poland, Slovakia, Iran, and Hungary, as reported by Arricau-Bouvery et al. (2006), Astobiza et al. (2012), Chmielewski et al. (2009), Roest et al. (2013), and Tilburg et al. (2012).

The genotyping of* C. burnetii* revealed the geog-raphic variability of the bacteria and helped to determine the origins of the epidemic (Szymanska-Czerwinska et al. 2019). Seven distinct genotypes have previously been identified in Spain (Astobiza et al. 2012), while another study conducted in Spain identified 15 different genotypes in samples obtained from milk tanks (Pinero et al. 2015). Moreover, Tilburg et al. (2012) applied the MLVA method and detected nine different genotypes in milk samples sold in the Netherlands. Another previous study found low-level differences in terms of the MLVA genotyping of bovine milk samples from Greece (Kalaitzakis et al. 2021). In Italy, the genotypes detected in both cow’s milk samples and goat’s milk samples were found to be different (Ceglie et al. 2015). In addition, in Poland, three different genotype groups have been reported (Szymanska-Czerwinska et al. 2019).

In conclusion, the detection of *C. burnetii*, which poses a risk to public health, in BTM reveals the necessity of routine implementation of reliable food systems in dairy enterprises. Moreover, the 16% positivity rate of *C. burnetii* in the BTM samples indicates a widespread agent in bovine dairy farms. In this study, two different genotypes were determined according to the number of studied samples, which indicates that geographically different genotypes may circulate. Conducting both prevalence and genotyping studies on BTM samples in Turkiye will contribute to determining the geographical distribution of the disease.
